# From genetic discovery to future personalized health research

**DOI:** 10.1016/j.nbt.2012.11.013

**Published:** 2013-03-25

**Authors:** Aarno Palotie, Elisabeth Widén, Samuli Ripatti

**Affiliations:** 1 The Wellcome Trust Sanger Institute, Cambridge, UK; 2 Institute for Molecular Medicine Finland (FIMM), University of Helsinki, Helsinki, Finland; 3 The Broad Institute of MIT and Harvard, Cambridge, MA, United States

## Abstract

During the past ten years the field of human disease genetics has made major leaps, including the completion of the Human Genome Project, the HapMap Project, the development of the genome-wide association (GWA) studies to identify common disease-predisposing variants and the introduction of large-scale whole-genome and whole-exome sequencing studies. The introduction of new technologies has enabled researchers to utilize novel study designs to tackle previously unexplored research questions in human genomics. These new types of studies typically need large sample sizes to overcome the multiple testing challenges caused by the huge number of interrogated genetic variants. As a consequence, large consortia-studies are at present the default in disease genetics research. The systematic planning of the GWA-studies was a key element in the success of the approach. Similar planning and rigor in statistical inferences will probably be beneficial also to future sequencing studies. Already today, the next-generation exome sequencing has led to the identification of several genes underlying Mendelian diseases. In spite of the clear benefits, the method has proven to be more challenging than anticipated. In the case of complex diseases, next-generation sequencing aims to identify disease-associated low-frequency alleles. However, their robust detection will require very large study samples, even larger than in the case of the GWA-studies. This has stimulated study designs that capitalize on enriching sets of low-frequency alleles, for example, studies focusing on population isolates such as Finland or Iceland. One example is the collaborative SISu Project (Sequencing Initiative Suomi) that aims to provide near complete genome variation information from Finnish study samples and pave the way for large, nationwide genome health initiative studies.

During the ten years that the ESF Functional Genomics program has existed, our thinking and tools to improve the understanding of the genetic background of diseases has transformed substantially. In 2002, when the program started, the first draft of the human genome had just been launched. Trying to understand the genetic background of common diseases, our thinking at that time was heavily influenced by our previous success to identify gene mutations causing Mendelian (monogenic) diseases. The thought was that similar family-based positional cloning strategies used to pinpoint genes for Mendelian diseases, would also be powerful in case of complex traits. However, the underlying genetic architecture of complex diseases turned out to be fundamentally different from those in classical Mendelian diseases thereby needing different study designs and tools. The vision for complex disease genetics was highlighted already in 1996 when Risch and Merikangas [1] pointed out that association studies are far more powerful than linkage studies in detecting multiple variants with small effect sizes. However, at that time, our tools were not yet sufficiently developed to implement these visions in practice. During the past ten years, that is, during the lifetime of the ESF program, the tools and opportunities have developed dramatically; the most important contributions being the drop in the cost of genotyping and sequencing.

As family-based linkage studies had modest success in identifying variants associated with the pathogenesis of common diseases, the contrary is true for the genome-wide association (GWA) studies. The work of the International HapMap consortium created a map of sequence variants common in the population (http://hapmap.ncbi.nlm.nih.gov/) that the industry used to develop standardized, cost-effective genotyping platforms. This enabled the genotyping of hundreds of thousands of variants in each individual study subject. Already the first GWA-studies clearly showed that the GWA-strategy is a successful method to detect associations between common polymorphisms and complex diseases and traits. As of July 2012, the GWA-study catalogue (http://www.genome.gov/gwastudies/) lists 1324 publications reporting robust associations between 6735 SNP markers and hundreds of diseases and traits [2].

By contrast, it also quickly became clear that most of the associated variants have very modest effect sizes (Odds ratios between 1.1 and 1.4). This inevitably means that GWA-studies need large sample sizes; samples from the low thousands up to hundreds of thousands are needed to pinpoint true associations among hundreds of thousands of markers with high statistical power. The need for large sample sizes beyond what individual study sites are able to collect quickly changed the mode of how genetic research is carried out. This new mode follows the global, collaborative pattern initially set by the Human Genome Project. No clinical center can work by them selves anymore. The groups had, and still have, to combine their resources to achieve meaningful statistical power. This was a major paradigm change in how academic research in this field is carried out. The necessity of large collaborative studies also had its impact on the funding structure. Large studies are expensive, beyond the reach of traditional academic funding modules, for example, the RO1 mechanism in US. Also in Europe, national and EU frameworks had a hard time (and still have) to respond to this quick change.

## Lessons from GWA studies to guide us to design sequencing studies

One important lesson from the success of the GWA-era was that it is crucial to carefully and systematically build large collaborative networks. For example, the early collaboration between the academic HapMap scientists and the genotype reagent industry was a key-element that drove the development of standardized genotyping platforms that the research field needed. These commercial genotyping chips were subsequently used in laboratories around the world, which enabled straightforward combination of data for subsequent meta-analyses.

Another lesson was that a solid statistical framework for study design and data analysis should be developed early on. In the GWA-studies, this consisted of strategies to control for genotype measurement errors, standardized approaches to control for confounding due to differential allele frequencies across populations and the multiple testing burden. It also included strategies for replication and validation of associations. As a result, the GWA-studies have implicated an avalanche of novel candidate genes and regions for functional studies. The well-defined statistical framework saved the field from a lot of unnecessary confusion. Now, when we have moved towards sequence-based low-frequency variant association studies, the benefits for setting rigorous statistical framework early on should be kept in mind.

## From the GWA locus to function

GWA-studies have been criticized for that they only provide information on genetic loci but no functional insight. Although we think that the critique in part is unjustified, it nonetheless highlights an important next step of the research. However, at present the field of functional biology is lacking efficient tools to shed more light on the function of the associated variants. One challenge is that the effect size of each individual variant is small and thus it is not trivial to develop readouts for cell- or model organism-based assays that reliably monitor small changes, e.g. in gene expression or phenotypes. Knock out assays may be a practical approach to elucidate gene function. Yet it is not an optimal model to decipher the in vivo consequence of genome variation with modest functional consequences. Secondly, the associated variants do not work in isolation. In some cases there is evidence that some cumulative effects of variants are additive [3], but there is also evidence that some variants can compensate for the effect of others [4]. Thirdly, most of the associated loci are located outside the coding regions of genes. Our understanding of the function and relevance of these intergenic, probably regulatory, regions is limited. This limited knowledge certainly limits the design of follow-up experiments. Large collaborative initiatives such as the ENCODE (Encyclopedia of DNA Elements) project aim to improve our understanding of the function of the genome. It provides new genome-wide tools to link disease-associated gene variants to gene function. One example is the open chromatin assays, including mapping of DNAase I hypersensitive sites in a large panel of cell lines (http://www.genome.gov/10005107).

## Towards a more complete understanding of the human genome variation

The success of the GWA-studies stimulated the field to proceed to provide an even more comprehensive understanding of the association of disease risk with genome variations. The introduction of new sequencing technologies and the dramatic drop of sequencing costs made it possible to explore the human variation in more detail. The first international effort was the 1000 genome project that aims to establish an extensive catalogue of human variations from 25 populations (www.1000genomes.org). The 1000 genomes project consists of anonymous participants without any phenotype information. Thus its main aim is to provide an international open access resource that serves as a basis for subsequent phenotype related studies. The 1000 genome project has been followed by large disease-specific sequencing studies, for example, the UK10K (www.UK10K.org) and GoT2D (Genetics of Type 2 Diabetes).

## Medical genome- and exome sequencing

The excitement of low cost sequencing has stimulated a boom of whole-genome and whole-exome sequencing studies. There are several successful examples of the identification of Mendelian gene mutations using new sequencing techniques (for references see [5]). At the same time, the challenges of this approach have also become evident. The interpretation of the sequence data and identification of functional disease causing mutations from more than 20,000 variants in each exome sample is not trivial. The interpretation of homozygote recessive mutations is typically the most straightforward. Even that is not always easy. However, the situation becomes much more complicated when we search for dominant acting variants, even if they were highly penetrant mutations. Each of us carries on an average 100 loss-of-function variants in our genome, 20 of which are in homozygous form [6]. We have very little understanding which of them, if any, may contribute to a phenotype. Thus detecting a loss-of-function variant in one sample has limited (if any) power to prove causality. The situation becomes even more complicated if the high-penetrant variant is a missense variant; each of us carries thousands of missense variants [5].

Some of the early attempts to identify Mendelian mutations using exome- or whole-genome sequencing were driven by high enthusiasm and sometimes not fully appreciating the statistical challenges of the study. Back in 1990s and early 2000, at the peak of positional cloning studies of Mendelian traits, rigorous standards were used before a mutation could be declared as disease-causing. Typically, the chromosomal location was first pinpointed by linkage, applying generally agreed significance thresholds. If a variant in the linked region was not seen in the background population, the same or different variants in the same gene had to be associated with the same phenotype in replication studies. Thirdly, at least some functional data had to be presented to convince the field, and the reviewers, that this variation/mutation was associated with the phenotype. It is hard to think of a reason why a more relaxed framework should be applied in exome sequence-based variant identification.

## Identification of low frequency variants contributing to common diseases

The identification of lower frequency variations (range 0.5–5% frequency in the general population) contributing to common diseases is expected to further expand our knowledge of the genetic background of these traits. One could expect that low-frequency variants will shed light especially on our understanding of disease mechanisms involved in the tails of a disease distribution. However, if we assume that the penetrance (risk ratio) of very low frequency alleles is modest and possibly only slightly higher than in the case of common variants (e.g. RR 1.2–2.0), the study size needed to achieve sufficient power is enormous. Given that it is complicated to reach significance in Mendelian traits, the challenge is even harder in case of common diseases [5]. Yet, discoveries such as the identification of low-frequency variants associating with sick sinus syndrome, gout and protection of Alzheimer's disease in the Icelandic population prove that with appropriate study designs and rigorous statistical methods in low/rare-variant disease-association studies can be very rewarding [7–9].

## Identification of de novo mutations

Because of the above-described challenges, study designs to filter the number of potential variants contributing to disease have been developed. One of them is to look for de novo mutations using a trio-design, that is, by sequencing the affected offspring and both parents. On an average each of us carries about 0.8–1.3 de novo mutations in our exome, not detectable in parents (the human genome mutation rate has been estimated to be 1.1–3 × 10^−8^ in a haploid genome per base per generation [10]). The trio-design has stimulated studies especially in the field of neurodevelopmental traits [11–13]. These results have provided a list of candidate genes that are awaiting future validation. In more than 900 autism trio-studies one consistent finding is that while the number of de novo events is not higher in affected autism offspring in comparison with control subjects, the ratio of nonsense versus missense variants is clearly higher in autism trio-cases. So far, trio-studies have highlighted the need for even larger sample sizes, even in a design where the number of candidate variants can be efficiently filtered down by only focusing on de novo events.

## Random population-based and family-based study designs

**Figure 1:**
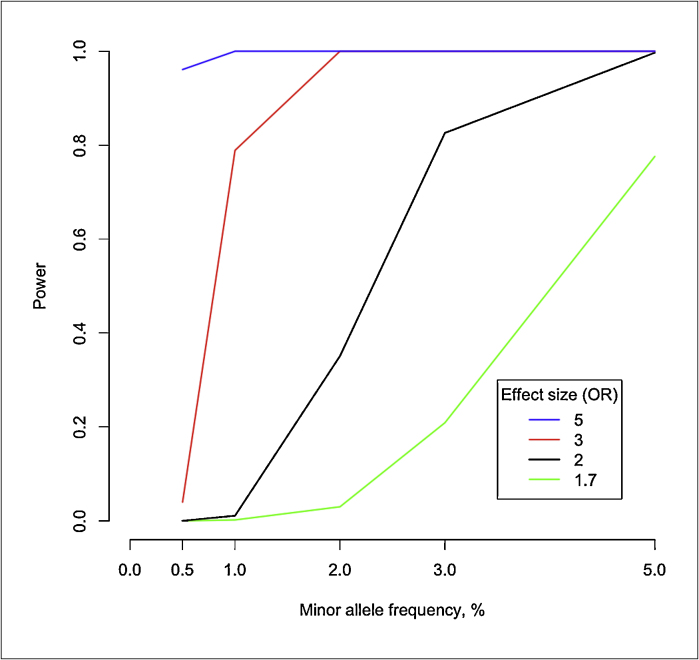
Statistical power to detect association between various low frequency variants, effect sizes, and disease for sample size of 2000 cases and 2000 controls. *P* < 5 × 10^−8^ is assumed for genome-wide significant association.

**Figure 2:**
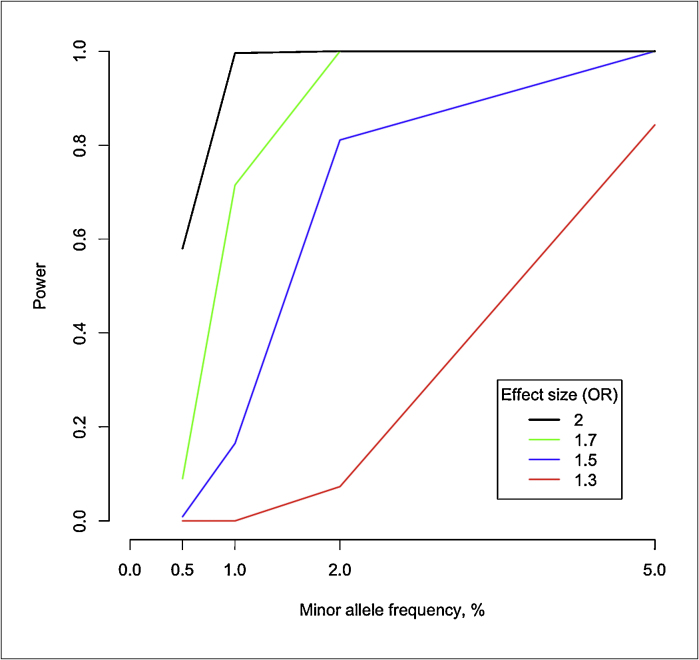
Statistical power to detect association between various low frequency variants, effect sizes, and disease for sample size of 10 000 cases and 10 000 controls. *P* < 5 × 10^−8^ is assumed for genome-wide significant association.

There is high interest in identifying also other types of rare disease-predisposing variants than de novo mutations. Studies utilizing both family and case–control designs are in progress. These include large studies such as the UK10K project that aims to sequence 4000 low-coverage whole genomes from the UK-Twin and ALSPAC cohorts and 6000 exomes from patients with selected diseases, including extreme obesity, ten different rare diseases, autism and schizophrenia. When writing this, the project is still in progress and it is still too early to evaluate its success. The data are not sufficient to guide the estimation of risk ratios associated with low-frequency (population frequency 0.5–5%) and rare (<0.5%) variants. However, it remains unlikely that there are many high-effect size variants contributing to the disease susceptibility in complex traits. If we speculate that typical effect sizes range between 1.2 and 3.0, we can foresee a need for sample sizes that are even bigger than in GWA-studies. Figures 1 and 2 demonstrate the statistical power to detect associations between low-frequency variants and disease for different allele frequencies and effect sizes. In Fig. 1, a sample size of 2000 cases and 2000 controls is assumed. Similarly, in Fig. 2, 10,000 cases and 10,000 controls are assumed.

Family-designs have been suggested to provide benefits and shortcuts also in sequencing studies of complex traits. The hypothesis is that an excess of disease susceptibility variants are clustered and more frequent in families with a specific disease than in the control population. Only a few family members need to be fully sequenced, whereas the remaining relatives can be more sparsely genotyped and the full genome variation can be imputed. The segregation of a disease-associated haplotype can then be traced in the full pedigree. Yet, the optimal statistical family-based analysis in complex traits is not fully worked out. The classical linkage analysis that emerged from a highly penetrant Mendelian type of mutation scenario might be too simplistic for complex traits. As stated above, the functional interplay of variants (variants might have different effects in different genetic backgrounds) might be complicated, resulting in substantially decreased power in transmission-based analyses. Thus it is probable that also family-based analyses will need large sample sizes to achieve statistically robust results. Recent studies from Iceland have demonstrated that extending the pedigree-design to ‘mega pedigree approaches’ and imputation using genealogical records or long-range haplotype matching can be highly effective [14].

## Population isolates

An extension of the ‘mega pedigree’ concept is to use population isolates to identify low-frequency and rare disease-causing alleles. Because of bottleneck effects, genetic drift and population expansion some rare alleles are enriched in population isolates like Finland. The hypothesis is that some of these alleles, which are extremely rare (population frequency < 0.1%), for example, in most European populations, have been enriched to frequencies between 1 and 5% in the population isolate. Some of the enriched variants might contribute to common diseases. Even though they could be neutral in an environment where the founder population was established more than thousand years ago, they may contribute to diseases in the modern lifestyle and environment. An enrichment of low-frequency alleles in the study population should boost the power significantly compared to more mixed populations. Therefore, the expectation is that smaller discovery sets are needed to achieve significant association in population isolates.

Additional to the benefits provided by the population structure, the Scandinavian health records, population cohorts and clinical sample collections are unique. These facts have stimulated several individual large whole-exome and whole-genome sequencing projects, which have organized themselves in a collaborative initiative called SISu (Sequencing Initiative Suomi (Suomi is the Finnish name for Finland)). By the end of 2012 we expect to have completed the sequencing of 8000 Finnish genomes or exomes. In addition, genome-wide association (GWA) data are available for about 40 000 Finns, complemented in many cases by low frequency variant genotype data (e.g. cardiometabo and exome chip). In a population isolate, like Finland, we expect that this will provide an exceptional imputation framework. Using this combined genome information we expect to impute a near complete genome variant set, enabling large-scale, low-cost custom genotyping in the entire collection of 200 000 individual's samples stored in the National Biobank (www.nationalbiobanks.fi). A well-characterized collection of samples, with near complete genome variant information and rich phenotypic and health record information, would provide a unique data-resource and reference database for building tools for personalized/precision medicine.

## From sequences towards personalized medicine

High throughput omics techniques provide opportunities to envision new medical and health care paradigms. Evidence-based medicine, which lies at the heart of current western approaches to healthcare, relies mainly on the statistical interpretation of data from large clinical trials. Although this is a well-tested strategy that will continue to inform medical practice, it is limited by a general failure to take into account more than a few personalized indicators such as, for example, sex, age, weight and smoking status. Therapeutic decisions that currently are based on average values from large studies, irrespective of a patient's individual characteristics and treatment strategies are therefore not always effectively targeted. Great hopes are being invested in personalized and stratified medicine as a form of health care that is better tailored to meet the individuals’ needs. An essential component of this aim is that complete individual genome sequences become a component of future clinical decision-making. Such projects as the SISu initiative would contribute by providing large and well-characterized reference datasets for implementing genomics in future clinical settings. The advantage of the individual sequence is that it, for many purposes, needs to be determined only once, and can be used multiple times over the life-course of an individual for diagnostic and therapeutic applications. The dramatically decreasing cost of sequencing will make sequencing only a minor fraction of average lifetime health costs. As a result, cumulative savings can be achieved over time.

Analyses of the complete spectrum of genome variants will create new analytical challenges and study design requirements. As stated above, tackling of these challenges requires large, well-documented data collections so that a large spectrum of risk combinations can be tested. Special populations provide specific opportunities to pioneer this task, and the Nordic countries provide unique advantages for these study designs. Yet, the procedures integrating very complex multilayer data and the development of tools that are helpful in clinical decision-making and health care need a lot of research.
